# Design, Characterization, and Lead Selection of Therapeutic miRNAs Targeting Huntingtin for Development of Gene Therapy for Huntington's Disease

**DOI:** 10.1038/mtna.2016.7

**Published:** 2016-03-22

**Authors:** Jana Miniarikova, Ilaria Zanella, Angelina Huseinovic, Tom van der Zon, Evelyn Hanemaaijer, Raygene Martier, Annemart Koornneef, Amber L Southwell, Michael R Hayden, Sander J van Deventer, Harald Petry, Pavlina Konstantinova

**Affiliations:** 1 Department of Research & Development, uniQure, Amsterdam, the Netherlands; 2 Department of Gastroenterology and Hepatology, Leiden University Medical Center, Leiden, the Netherlands; 3 Centre for Molecular Medicine and Therapeutics, University of British Columbia, Vancouver, Canada

**Keywords:** adeno-associated virus, gene silencing, humanized HD mouse model, Huntington's disease, therapeutic microRNAs

## Abstract

Huntington's disease (HD) is a neurodegenerative disorder caused by accumulation of CAG expansions in the huntingtin (*HTT*) gene. Hence, decreasing the expression of mutated HTT (mtHTT) is the most upstream approach for treatment of HD. We have developed HTT gene-silencing approaches based on expression cassette-optimized artificial miRNAs (miHTTs). In the first approach, total silencing of wild-type and mtHTT was achieved by targeting exon 1. In the second approach, allele-specific silencing was induced by targeting the heterozygous single-nucleotide polymorphism (SNP) rs362331 in exon 50 or rs362307 in exon 67 linked to mtHTT. The miHTT expression cassette was optimized by embedding anti-HTT target sequences in ten pri-miRNA scaffolds and their HTT knockdown efficacy, allele selectivity, passenger strand activity, and processing patterns were analyzed *in vitro.* Furthermore, three scaffolds expressing miH12 targeting exon 1 were incorporated in an adeno-associated viral serotype 5 (AAV5) vector and their HTT knock-down efficiency and pre-miHTT processing were compared in the humanized transgenic Hu128/21 HD mouse model. Our data demonstrate strong allele-selective silencing of mtHTT by miSNP50 targeting rs362331 and total HTT silencing by miH12 both *in vitro* and *in vivo*. Ultimately, we show that HTT knock-down efficiency and guide strand processing can be enhanced by using different cellular pri-miRNA scaffolds.

## Introduction

Huntington's disease (HD) is a rare, fatal, neurodegenerative genetic disorder that affects muscle coordination and leads to behavioral symptoms and cognitive decline, resulting in total physical and mental deterioration over a 12- to 15-year period.^1^ HD is caused by the expansions of the polyglutamine (polyQ) tract in the multifunctional huntingtin protein (HTT). Despite the ability to identify HD mutation carriers decades before onset, there is currently no available therapy that can delay onset or slow progression of the disease.

The monogenetic etiology, autosomal dominant inheritance, and the fully penetrant phenotype following a long pre-syndromatic phase place HD as a good candidate for gene therapy. Consequently, there is a wide variety of potential HD therapeutic strategies aiming at delivery of neurotrophic factors, activation of neuronal stem cells, Ca^2+^ and mitochondrial function normalization and mutant (mt)HTT targeting.^2^ The preferred strategy by many groups including ours is direct lowering of the *mtHTT* mRNA level.

Interfering with *HTT* mRNA expression has been widely explored using synthetic antisense oligonucleotides (ASOs), single-stranded RNAs (ssRNAs), small interfering RNAs (siRNAs), expressed short hairpin RNAs (shRNAs), or artificial microRNAs (miRNAs).^3,4,5^

ASO and siRNA are chemically modified DNA or RNA oligos, respectively that bind to the *HTT* transcript. ASO-mediated HTT lowering has been successfully shown in murine HD models and holds a great promise for HD therapy.^6,7^ Infusion of siRNAs targeting HTT into the striatum of nonhuman primates has demonstrated significant silencing of HTT in a dose-dependent manner.^8^ However, it still needs to be determined if sufficient ASO and siRNA biodistribution, efficacy, and safety in the large animal and human brain can be achieved. Moreover, the relatively short half-life of the ASO and siRNA would require repeated administration presenting clinical burden for the HD patients.

Expressing siRNAs from shRNA or miRNA scaffolds has shown great promise for HTT lowering in murine HD models when delivered with adeno-associated viral (AAV) vectors.^4,9,10^ The shRNA- or miRNA-expression can be driven by polymerase (pol) III or pol II promoters allowing for tissue- or cell-specific expression.^11^ Moreover, the expressed shRNA or miRNA provide long-term silencing of the *HTT* transcript, avoiding the necessity for readministration of the therapeutic.

Gene silencing may be indiscriminative between the wild type (wt) and mtHTT or can selectively silence only the mutated allele with expanded CAG repeats. Multiple lines of evidence indicate that total silencing of HTT can be performed in a certain therapeutic window.^12^ Two studies used AAV2-miHTT or AAV2-shHTT to partially silence wtHTT in a healthy rhesus macaque putamen. In both studies, 45% reduction of HTT did not induce any abnormal motor phenotype, altered circadian behavior, fine motor skill deficits, neuronal loss, gliosis, or immune response at 6 weeks or 6 months postinjection, respectively.^13,14^ Selective silencing only of the mtHTT presents challenges as the difference from the wtHTT allele is mainly in the higher number of CAG repeats. Allele-specific HTT silencing via targeting of the CAG repeat is possible using ssRNAs and can be specific to only the mutant allele.^15^ Single-nucleotide polymorphism (SNP) genotyping of HD patients has identified ~41 SNPs heterozygous in at least 30% of the patients.^16^ Those SNPs are good targets for silencing of only the mtHTT while maintaining the expression of the wtHTT allele. Allele-selective mtHTT silencing has been achieved with ASOs, siRNAs, shRNAs, or miRNAs directed toward heterozygous SNPs in exons 39 (rs363125), 50 (rs362331), 60 (rs2276881), or 67 (rs362307).^7,16,17,18,19,20^

Despite the existence of many algorithms and rules for design of therapeutic miRNAs targeting either both HTT alleles or only the mutant allele based on SNPs, the evaluation of efficacy and allele selectivity of every molecule still needs to be performed empirically. In the current study, we designed artificial miRNAs targeting human HTT exon 1 (miH), the expanded CAG repeats (miCAG), C or T isoform of SNP rs362331 in exon 50 (miSNP50C and miSNP50T) and T isoform of SNP rs362307 in exon 67 extended to 3'UTR (miSNP67T). miH12 variant silenced efficiently both wt and mtHTT reporters, while miCAG constructs failed to induce allele-specific silencing. The latter was achieved by miSNP50 and miSNP67 variants bearing secondary mismatches to the wtHTT. Furthermore, to reduce the chance of off-target effects due to passenger strand activity, artificial miRNA processing was “biased” towards high concentrations of guide strand production by using different cellular pri-miRNA scaffolds. This resulted in differential guide and passenger strand processing patterns revealed by next-generation sequencing (NGS) and HTT knockdown efficiency *in vitro.* Ultimately, to compare the HTT silencing efficiency and miH12 processing *in vivo*, three AAV5-miH12 vectors were generated and tested in the humanized Hu128/21 HD mouse model. For the first time, we show that the therapeutic pri-miRNA scaffold influences HTT knock-down and guide strand processing *in vivo*. The best therapeutic scaffolds were miH12-451, miSNP50C-451 and miSNP50T-451 as they induced strong HTT silencing and had almost undetectable passenger strand concentration with no activity.

## Results

### Huntingtin (*HTT*) target sequences and design of artificial microRNA-expression constructs

**Figure 1:**
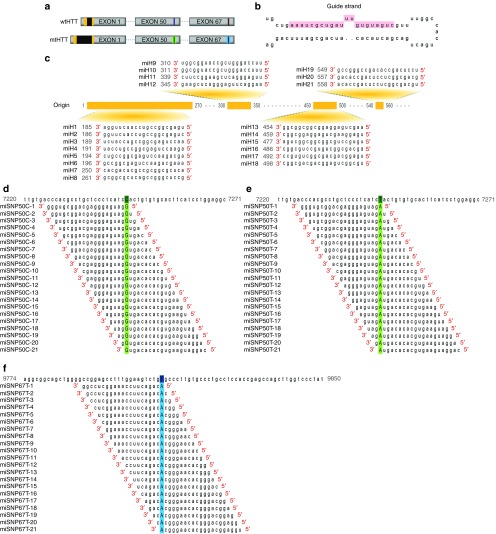
**Design of therapeutic miHTT variants for Huntington's disease (HD) therapy**. (**a**) Schematic of exons 1, 50, and 67 (extended to 3'UTR) of human wild-type (wt) or mutated (mt) huntingtin (HTT) gene (gb|L12392.1|HUMHDA) with CAG expansions in black, target sequences for miH1-miH21 in yellow, miSNP50 variants in green, and miSNP67 variants in blue. Single-nucleotide polymorphisms (SNPs) located either in exon 50 (rs362331) or exon 67 extended to 3'UTR (rs362307) are represented as dark stripes within the target regions. (**b**) The engineered mmu-pre-miR-155 scaffold used for miHTT expression with highlighted 21 nt guide strand. (**c**) Twenty-one miHTT variants were designed to target HTT exon 1. In a consecutive manner, miH1-miH21 bind to HTT exon 1 shown in yellow with the target starting nucleotide positions in gray. (**d**) miSNP50C variants targeting the C isoform of mtHTT-associated SNP rs362331 in exon 50. Micro-shifting of mature miSNP50C sequences along the C isoform generated 21 variants with the SNP-matching position 1–21 (miSNP50C-1—miSNP50C-21). The C isoform is represented in dark green and the SNP-matching nucleotide in light green. (**e**) miSNP50T variants targeting the T isoform of mtHTT-associated SNP rs362331 in exon 50. miSNP50T variants were generated similarly to miSNP50C. (**f**) miSNP67T variants targeting the T isoform of mtHTT-associated SNP rs362307 in exon 67. miSNP67T variants were generated similarly to miSNP50T. The T isoform of rs362307 is represented in dark blue and the SNP-matching nucleotide in light blue. A list of all pre-miHTT scaffolds can be found in **Supplementary Table S1**.

Three regions in exon 1, 50, and 67 (extended to 3'UTR) of the human *HTT* gene were selected to design artificial anti-*HTT* miRNAs (miHTTs) (**Figure 1a**). All miHTT variants were generated to specifically target the human *HTT* transcripts. The miHTT sequences were embedded in the engineered mmu-pre-miR-155 scaffold (**Figure 1b**). The first miHTT group, miH1-miH21, was developed to target sequences within exon 1, hence to induce HTT knock-down of both wt and mt alleles (**Figure 1c**). An alternative strategy aims at suppressing only the mtHTT allele. The majority of HD patients are heterozygous for the CAG expansion and the selective targeting of the longer HD-associated CAG tract could potentially treat all patients.^21^ Therefore, we designed miCAG1—miCAG15 targeting the CAG repeats in exon 1 to selectively suppress only the mtHTT allele. Recently, several SNPs have been identified in high linkage disequilibrium with the disease-associated allele.^3,17,20,22^ Notably, heterozygocity of SNP rs362307 located in 3'UTR of *HTT* (the extended exon 67) was observed in 48.6% of US and European HD representative population and the T isoform of rs362307 constituted 26%. In the same study, heterozygocity (C/T) of SNP rs362331 located in exon 50 was observed in 39.4%.^17^ In another study, heterozygocity (C/T) of rs362331 was observed in 47.6% of Italian HD representative population.^20^ Therefore, in pursuit of selective degradation of the mutant transcript from the HD allele, we selected HTT sequences that contain HD-related isoforms C or T of rs362331 in exon 50 and the isoform T of rs362307 located in exon 67 (extend to 3'UTR). Consequently, we designed miSNP50C-1—miSNP50C-21 to silence the transcripts carrying the C isoform of rs362331 (**Figure 1d**), miSNP50T-1—miSNP50T-21 to suppress the transcripts with the U isoform of rs362331 (**Figure 1e**), and miSNP67T-1—miSNP67T-21 to target transcripts with the U isoform of rs362307 (**Figure 1f**).

### *In vitro* silencing efficacy of artificial miHTT constructs

**Figure 2:**
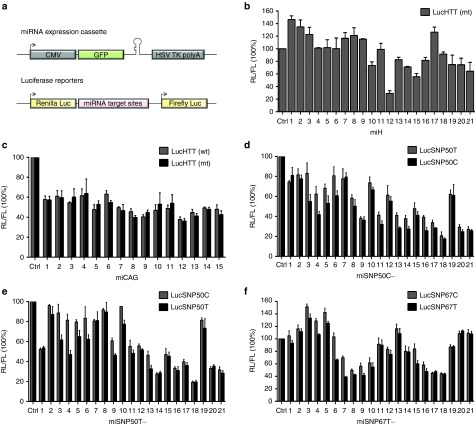
**Luciferase reporter knock-down by different miHTT variants**. (**a**) Schematic representation of pcDNA6.2-CMV-GFP-miHTT constructs and luciferase (Luc) reporters. Green fluorescent protein (GFP) and miHTT variants were expressed as one transcript from the cytomegalovirus (CMV) promoter and the expression cassette terminated with the herpes simplex virus thymidine kinase polyadenylation (HSV TK polyA) signal. For the Luc reporters, sequences from HTT exon 1 with 19 or 73 CAG repeats (LucHTT(wt) or LucHTT(mt), respectively), exon 50 (LucSNP50C, LucSNP50T), or exon 67 (LucSNP67C, LucSNP67T) were cloned behind the renilla luciferase (RL) gene. In addition, firefly luciferase (FL) was coexpressed from the vector as an internal control. (**b**) LucHTT(mt) reporter knock-down by miH1-miH21 constructs targeting sequences in HTT exon 1. Human embryonic kidney (HEK)293T cells were cotransfected with 50 ng Luc reporter and 50 ng miH1-miH21 constructs. RL and FL were measured 2 days post-transfection and RL was normalized to FL expression. Scrambled (Ctrl) served as a negative control and was set at 100%. (**c**) LucHTT(wt) and LucHTT(mt) reporter knock-down by 15 miCAG constructs targeting the CAG repeats of exon 1 measured 2 days post-transfection. The experimental setup is as described in (**b**). (**d**) LucSNP50 reporter knock-down by miSNP50C-1—miSNP50C-21. LucSNP50C and LucSNP50T expressed a partial sequence of exon 50 with the isoform C or T of rs362331, respectively. All miSNP50C variants perfectly match to LucSNP50C and have 1 nt mismatch with LucSNP50T target sequences. HEK293T cells were cotransfected with 100 ng Luc reporter and 10 ng miSNP50C constructs. Luciferase expression was measured as described in (**b**). (**e**) LucSNP50 reporter knock-down by miSNP50T-1—miSNP50T-21 constructs. All miSNP50T variants perfectly match to LucSNP50T and have 1 nt mismatch with LucSNP50C target sequences. Transfections and luciferase measurements were performed as described in (**d**). (**f**) LucSNP67 reporter knock-down by miSNP67T-1—miSNP67T-21 constructs. LucSNP67C and LucSNP67T reporters expressed a partial sequence of exon 67 with the isoform C or T of rs362307, respectively. All miSNP67T variants perfectly match with LucSNP67T and have 1 nt mismatch with LucSNP67C target sequences. Transfections and luciferase measurements were performed as in (**b**). The luciferase knock-down are representative figures of three independent experiments.

To evaluate the HTT knock-down efficacy by the miHTT-expression constructs *in vitro*, we cotransfected them in human embryonic kidney (HEK)293T cells with luciferase (Luc) reporters bearing complementary HTT regions fused to the renilla luciferase (RL) gene. Independently from RL, firefly luciferase (FL) was expressed from the reporter vector to correct for transfection efficiency (**Figure 2a**). From miH1-miH21 constructs designed to target both HTT alleles, miH10, miH14, miH15, miH19, miH20, and miH21 induced mild knock-down between 25–45% of LucHTT(mt) expressing a portion of human *HTT* exon 1 encompassing 73 CAG repeats (**Figure 2b**). miH12 construct was highly effective and induced more than 70% inhibition of LucHTT(mt) reporter.

For the allele-specific HTT suppression based on discriminating between different lengths of the CAG expansion, we evaluated the silencing efficacy of 15 miCAG constructs on Luc reporters expressing either 19 or 73 CAG repeats, named LucHTT(wt) and LucHTT(mt) respectively (**Figure 2c**). In contrary to previously published studies using siRNAs, ss-siRNAs, ASOs, or different artificial miRNAs, all miCAG variants showed poor allele selectivity and neither of the constructs induced HTT knock-down stronger than 60%.^15,19,23,24,25^

Previously, several small molecules such as siRNAs, ASOs, shRNAs, and different artificial miRNAs targeting the isoforms of rs362307 or rs362331 were designed and experimentally tested for the allele-specific silencing of mtHTT allele.^16,17,19,20^ To estimate the ability of our miHTT constructs to induce the allele selectivity by owning only one nucleotide difference between the match and mismatch target sequences, we first cotransfected miSNP50C constructs with LucSNP50C or LucSNP50T reporters carrying target sequences of exon 50 with either C or T isoform of rs362331, respectively (**Figure 2d**). Seven miSNP50C variants with the SNP-matching nucleotide at positions 13, 14, 16, 17, 18, 20, and 21 induced mtHTT knock-down stronger than 70% with miSNP50C-18 being the most efficient and inducing ±80% knock-down of both C and T target variants. Neither of these constructs showed great potential to discriminate between the match and mismatch targets. Similarly to the miSNP50C constructs, cotransfections of the miSNP50T constructs with the match LucSNP50T and mismatch LucSNP50C reporters showed strong HTT inhibition by a number of variants but overall poor allele selectivity (**Figure 2e**). Three miSNP50T variants with the SNP-matching nucleotide at positions 14, 18, and 21 induced HTT knock-down stronger than 70% with miSNP50T-18 being again the most efficient. Finally, miSNP67T constructs were cotransfected with the match LucSNP67T and mismatch LucSNP67C reporters. miSNP67T constructs with the SNP-matching nucleotide at position 7, 8, 9, 16, 17, and 18 induced mild 50–60% knock-down of their match target and poor allele selectivity (**Figure 2f**). The most efficient was miSNP67T with the SNP-matching nucleotide at position 7 and showed HTT knock-down of ±60% with mild allele selectivity. Based on these results, miH12, miSNP50C-18, miSNP50T-18, and miSNP67T-7 were selected for further optimizations.

### Enhancement of allele selectivity by introduction of a single base substitution in miSNP50C-18, miSNP50T-18, and miSNP67T-7 constructs

**Figure 3:**
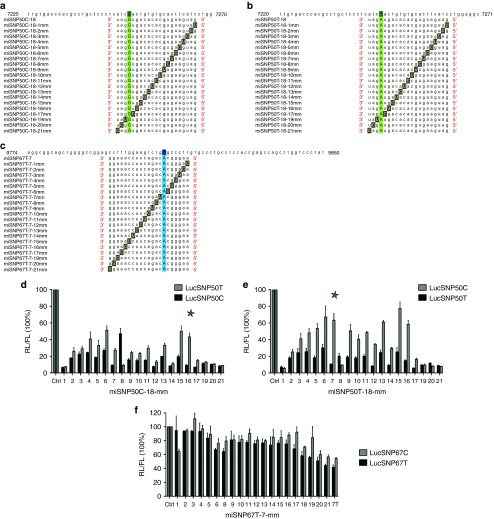
**Introduction of a mismatch to miSNP50C-18, miSNP50T-18, and miSNP67T-7 to improve mtHTT allele selectivity**. (**a**) Twenty variants named miSNP50C-18-1mm to miSNP50C-18-21mm were generated with one mismatch to the C-target sequence at positions 1–21 and two mismatches to the T-target sequence: one at position 18 and the other at positions 1–21. In all variants, G or C were replaced by T, and A or T by C. The C isoform of rs362331 is represented in dark green, the SNP-matching nucleotide in light green and the mismatch in grey. (**b**) Twenty variants named miSNP50T-18-1mm—miSNP50T-18-21mm were designed as described in (**a**). (**c**) Twenty variants named miSNP67T-7-1mm—miSNP67T-7-21mm were designed as described in (**a**). The T isoform of rs362307 is represented in dark blue, the SNP-matching nucleotide in light blue and the mismatch in dark gray. (**d**) LucSNP50 reporter knock-down by miSNP50C-18-1mm to miSNP50C-18-21mm constructs. Human embryonic kidney (HEK)293T cells were cotransfected with 50 ng Luc reporter and 50 ng miSNP50C-18 constructs. Renilla (RL) and firefly (FL) luciferases were measured 2 days post-transfection and RL was normalized to FL expression. Scrambled (Ctrl) served as a negative control and was set at 100%. (**e**) LucSNP50 reporter knock-down by miSNP50T-18-1mm to miSNP50T-18-21mm constructs. Transfections and luciferase measurements were performed as described in (**d**). (**f**) LucSNP67 reporter knock-down by miSNP67T-7-1mm—miSNP67T-7-21mm constructs. HEK293T cells were co-transfected with 30 ng Luc reporter and 3 ng miSNP67T constructs. Luciferase expression was measured as in (**d**). The miSNP50C-18-16mm and miSNP50T-18-7mm constructs selected for further optimization are indicated with the asterisk. The luciferase knock-down are representative figures of four independent experiments.

miSNP50C-18, miSNP50T-18, and miSNP67T-7 constructs showed good HTT silencing efficacy but poor allele selectivity *in vitro*. In order to enhance their potential to discriminate between two heterozygous HTT alleles, we introduced a single base change at each possible position of the guide strand while creating the mismatch with the target sequences.^26^ For each base substitution, G or C were replaced by T, and A or T were replaced by C. Since the inserted guide strand sequences of all three variants are 21 nt long with one crucial SNP-matching nucleotide, we generated 20 constructs for miSNP50C-18 (**Figure 3a**), miSNP50T-18 (**Figure 3b**), and miSNP67T-7 (**Figure 3c**).

To determine whether a single base substitution can improve the allele selectivity, we first cotransfected miSNP50C-18-1mm—miSNP50C-18-21mm with LucSNP50C or LucSNP50T reporter (**Figure 3d**). Each miSNP50C-18-mm construct acquired one nucleotide mismatch with LucSNP50C reporter and a second nucleotide mismatch with LucSNP50T reporter. Overall, we observed a variation between miSNP50C-18-mm constructs in both the reporter silencing efficacy and allele discrimination. miSNP50C-18-6mm induced mild allele selectivity and ±70% knock-down of the match LucSNP50C reporter. miSNP50C-18-15mm and miSNP50C-18-16mm showed good allele selectivity and match reporter knock-down stronger than 80% with miSNP50C-18-16mm being the most efficient.

Cotransfections of miSNP50T-18-1mm—miSNP50T-18-21mm with LucSNP50C and LucSNP50T reporters showed similar trend of match reporter knock-down and allele selectivity as with miSNP50C-18-mm variants, but a greater potential to discriminate between the two reporters (**Figure 3e**). miSNP50T-18-7mm and miSNP50T-18-16mm triggered good allele selectivity and match reporter knock-down stronger than 80% with miSNP50T-18-7mm being the most efficient.

Cotransfections of miSNP67T-7-1mm—miSNP67T-7-21mm with LucSNP67C and LucSNP67T showed overall poor enhancement of allele selectivity and a reduction of silencing efficacy compared with the initial miSNP67T-7 (**Figure 3f**).

### Generation and testing of miHTT expression vectors suitable for clinical use

**Figure 4:**
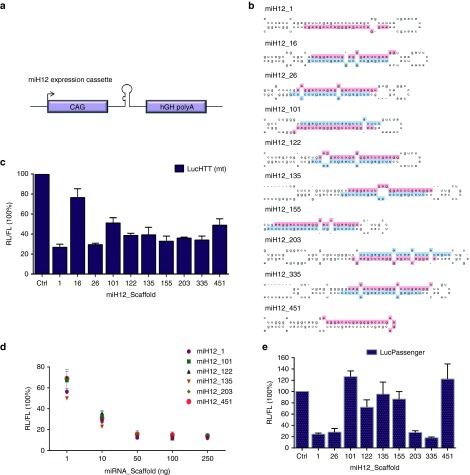
**Optimization of the miH12 expression cassette to address efficacy and passenger strand activity**. (**a**) Schematic representation of the pri-miH12 expression cassette with the CMV immediate-early enhancer fused to chicken β-actin (CAG) promoter and human growth hormone polyadenylation (hGH polyA) signal. (**b**) Ten cellular pri-miRNA scaffolds were selected from miRBase database (www.mirbase.org) based on the guide/passenger strand prevalence and RNA folding. Twenty-one nt of the guide strand were replaced by the mature miH12 sequence and the passenger strand was corrected in order to preserve pri-miH12 scaffolding. Based on miRBase, the predicted guide and passenger strand sequences of the cellular pre-miRNA scaffolds are indicated in red and blue, respectively. (**c**) LucHTT(mt) reporter knock-down by ten miH12 scaffolds. Human embryonic kidney (HEK)293T cells were cotransfected with 100 ng LucHTT(mt) reporter and 10 ng miH12 constructs. Renilla (RL) and firefly (FL) luciferases were measured 2 days post-transfection and RL was normalized to FL expression. Scrambled (Ctrl) served as a negative control and was set at 100%. (**d**) Titration curve of miH12 embedded in the human miR-1, miR-101, miR-122, miR-135, miR-203, and miR-451 scaffolds. HEK293T cells were cotransfected with 50 ng LucHTT(mt) reporter and 1, 10, 50, 100, or 250 ng of miH12 constructs. Luciferase expression was measured as described in (**c**). (**e**) *In vitro* passenger strand activity of miH12-1, miH12-26, miH12-101, miH12-122, miH12-135, miH12-155, miH12-203, miH12-335, and miH12-451 scaffolds. For the LucPassenger reporters, reverse complement sequences to the miH12 passenger strand sequences were cloned behind RL. In addition, FL was coexpressed from the vector as an internal control. Transfections and luciferase measurements were performed as in (**c**). The luciferase knock-down are representative figures of three independent experiments.

Thus far, all miHTT variants were expressed jointly with green fluorescent protein (GFP) from the cytomegalovirus (CMV) promoter. In a vision of clinical trial preparations for the total HTT knock-down, we chose to optimize further the expression cassette of miH12. First, the CMV promoter was replaced by an ubiquitous CAG promoter that consisted of cytomegalovirus immediate-early enhancer fused to chicken β-actin promoter (**Figure 4a**). This promoter is known to drive stable high expression of a transgene and to be highly effective in the brain.^27^ Second, GFP was excluded from the expression vector to eliminate the possibility of the host immune response. To address the influence of the pri-miRNA scaffold on the processing, silencing potential of miH12 and the passenger strand activity, the 21 nt guide sequence was embedded in the human miR-1–2, miR-16-1, miR-26a-1, miR-101-1, miR-122, miR-135b, miR-155, miR-203a, miR-335, miR-451a scaffolds with 200 nt 5' and 3' encompassing flanking regions (**Figure 4b**). The pri-miRNA scaffolds were selected based on their stem-loop structure and the high guide versus passenger strand ratios obtained from miRBase release 21 (http://www.mirbase.org/). The corresponding passenger strand (miH12*) was corrected in order to preserve the original pri-miRNA structure.

The silencing efficacy of ten CAG-miH12 expression constructs, named miH12-1, miH12-16, miH12-26, miH12-101, miH12-122, miH12-135, miH12-155, miH12-203, miH12-335, and miH12-451 was measured by the LucHTT(mt) knock-down in HEK293T cells (**Figure 4c**). Heterogeneity in the silencing effect was observed for the different constructs. A weak 20% HTT knock-down was induced by miH12-16 scaffold. miH12-101 and miH12-451 scaffolds triggered ±50% HTT knock-down while miH12-1, miH12-26, miH12-122, miH12-135, miH12-155, miH12-203, and miH12-335 scaffolds reached 60–70% HTT knock-down. To more precisely define the knock-down efficacy of the miH12 scaffolds, titration curves were generated for the best candidates (**Figure 4d**). Only at 1 ng of the transfected plasmid, we observed a clear difference in the LucHTT(mt) knock-down between individual scaffolds. With 50 ng and higher concentrations of the plasmid, all constructs showed more than 70% LucHTT(mt) knock-down.

It has been shown that thermodynamic properties of double-stranded miRNA molecules play key roles in strand selection and functional assymetry.^28,29,30^ To estimate the silencing activity of the complementary miH12* passenger strand *in vitro*, we generated Luc reporters carrying extended miH12* complementary sequences (LucPassenger). Cotransfections of LucPassenger constructs with the corresponding miH12 scaffolds in HEK293T cells showed variations in LucPassenger knock-down (**Figure 4e**). We detected no or low activity of the miH12* processed from miH12-101, miH12-122, miH12-135, miH12-155, and miH12-451 scaffolds. Strong LucPassenger knock-down was induced by the rest of the miH12 scaffolds.

### *In vitro* allele-selective HTT suppression by miSNP50C and miSNP50T processed from miR-1, miR-101, miR-135, and miR-451 scaffolds

**Figure 5:**
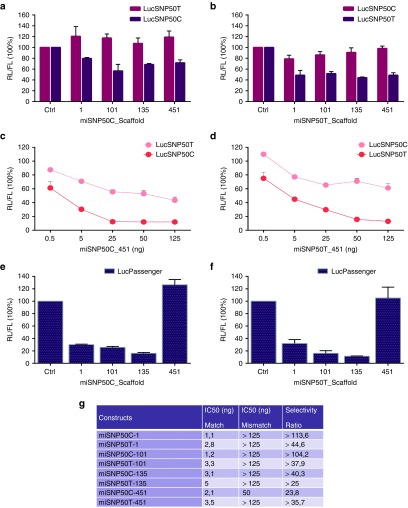
**Optimization of the miSNP50C and miSNP50T expression cassette to address efficacy and passenger strand activity**. (**a**) LucSNP50 reporter knock-down by four different pri-miSNP50C scaffolds. Twenty-one nt mature miSNP50C-18-16mm sequence, from now referred as miSNP50C, was embedded as a guide strand in the human miR-1, miR-101, miR-135, and miR-451 scaffolds and the passenger strand was corrected in order to preserve pri-miSNP50C folding. Human embryonic kidney (HEK)293T cells were cotransfected with 50 ng luciferase (Luc) reporter and 10 ng miSNP50C constructs. Renilla (RL) and firefly (FL) luciferases were measured 2 days post-transfection and RL was normalized to FL expression. Scrambled (Ctrl) served as a negative control and was set at 100%. (**b**) LucSNP50 reporter knock-down by four different pri-miSNP50T constructs. Twenty-one nt mature miSNP50T-18-7mm sequence, from now referred as miSNP50T, was embedded as a guide strand in the human miR-1, miR-101, miR-135, and miR-451 scaffolds and the passenger strand was corrected in order to preserve pri-miSNP50T folding. HEK293T cells were co-transfected with 50 ng Luc reporter and 10 ng pri-miSNP50T constructs. Luciferase expression was measured as in (**a**). (**c**) Titration curve of miSNP50C embedded in the miR-451 scaffold, from now referred as miSNP50C-451. HEK293T cells were cotransfected with 25 ng Luc reporter and 0.5, 5, 25, 50, or 125 ng of miSNP50C-451 construct. Luciferase expression was measured as described in (**a**). (**d**) Titration curve of miSNP50T embedded in the miR-451 scaffold, from now referred as miSNP50T-451. Transfections and luciferase measurements were performed as in (**c**). (**e**) *In vitro* passenger strand activity of miSNP50C embedded in the miR-1, miR-101, miR-135, and miR-451 scaffolds. For the LucPassenger reporters, reverse complement sequences to the four miSNP50C passenger strand sequences were cloned behind RL. In addition, FL was coexpressed from the vector as an internal control. HEK293T cells were cotransfected with 25 ng Luc reporter and 5 ng miSNP50C constructs. Luciferase measurements were performed as in (**a**). (**f**) *In vitro* passenger strand activity of miSNP50T embedded in the miR-1, miR-101, miR-135, and miR-451 scaffolds. Transfections and luciferase measurements were performed as in (**e**). (**g**) Allele selectivity ratios of miSNP50C and miSNP50T embedded in the miR-1, miR-101, miR-135, and miR-451 scaffolds. IC50 indicates a concentration (ng) of miSNP50 construct that is required for 50% Luc reporter knock-down. LucSNP50C represents a match reporter for miSNP50C constructs and a mismatch reporter for miSNP50T constructs, and vice versa. The allele selectivity ratios were calculated as IC50(mismatch)/IC50(match). The luciferase knock-down are representative figures of two independent experiments.

In a vision of clinical trial preparations for the allele-specific HTT knock-down by targeting the HD-associated SNP rs362331, we optimized further the expression cassette for miSNP50C-18-16mm (from now referred as miSNP50C) and miSNP50T-18-7mm (from now referred as miSNP50T). Based on *in vitro* results from ten miH12 scaffolds, we selected miR-1, miR-101, miR-135, and miR-451 scaffolds for miSNP50C and miSNP50T. miH12-135 was highly efficient and together with miH12-101 and miH12-451 did not produce any passenger strand. The miR-1 scaffold was included based on the simplicity of the stem structure. To evaluate their silencing and allele-selectivity potential, we cotransfected the different scaffolds with LucSNP50C and LucSNP50T reporters (**Figure 5a**,**b**). We observed a similar trend of match reporter knock-down and allele selectivity for all four scaffolds with miSNP50C-101 and miSNP50T-135 being the most efficient. Further titrations of the miSNP50 scaffolds revealed a concentration-dependent silencing. Representative figures of miSNP50C-451 and miSNP50T-451 titrations are shown (**Figure 5c,d,g**). Next, we addressed the passenger strand activity of the miSNP50C and miSNP50T scaffolds on LucPassenger reporters carrying extended miSNP50C* and miSNP50T* complementary target sequences. On the contrary to our results with miH12 constructs, we observed strong LucPassenger knock-down by miSNP50C* and miSNP50T* strands processed from miR-1, miR-101, and miR-135 scaffolds (**Figure 5e**,**f**). miR-451 was the only scaffold that did not induce LucPassenger reporter silencing.

### Expression of miH12 and miSNP50 from various cellular pri-miRNA scaffolds results in different guide and passenger strand processing

To demonstrate the influence of the cellular pri-miRNA scaffolds on miH12 and miSNP50 processing, we analyzed the prevalence and sequence composition of the guide and passenger strands by NGS. NGS was performed on small RNAs isolated from HEK293T cells transfected with miH12-1, miH12-101, miH12-122, miH12-135, miH12-155, miH12-203, or miH12-451 expression constructs. For miSNP50C and miSNP50T, we analyzed the processing from miR-135 and miR-451 scaffolds. For each sample, we obtained between 15–30 mln small RNA reads that were subsequently adaptor-trimmed and aligned against the corresponding reference sequence. All reads shorter than 10 nt, longer than 45 nt, and represented less than 10 times were excluded from the analysis.

**Figure 6:**
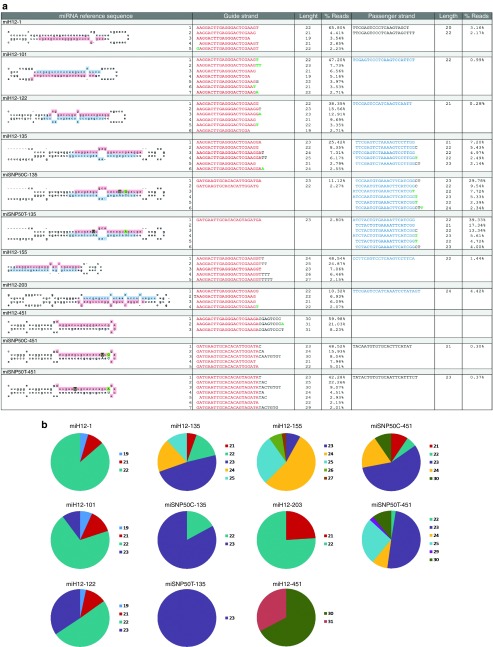
**Next-generation sequencing (NGS) analysis on the processing of miH12-1, miH12-101, miH12-122, miH12-135, miH12-155, miH12-203, miH12-451, miSNP50C-135, miSNP50T-135, miSNP50C-451, and miSNP50T-451 scaffolds**. (**a**) Sequence distribution (%) of reads mapping to miH12-1, miH12-101, miH12-122, miH12-135, miH12-155, miH12-203, miH12-451, miSNP50C-135, miSNP50T-135, miSNP50C-451, and miSNP50T-451 pre-miRNA scaffold sequences. Human embryonic kidney (HEK)293T cells were transfected with 4 μg of the constructs, 48 hours post-transfection RNA was isolated and small RNA NGS was performed. For each variant, the miHTT scaffold is shown in the first column. Based on miRBase, the predicted guide and passenger strand sequences of the cellular pri-miRNA scaffolds are indicated in red and blue, respectively, 5' and 3' flanking nucleotides in black, and a mismatch with the reference sequence in green. (**b**) Length distribution of reads mapping to miH12-1, miH12-101, miH12-122, miH12-135, miH12-155, miH12-203, miH12-451, miSNP50C-135, miSNP50T-135, miSNP50C-451, and miSNP50T-451 scaffold sequences. For the read alignments, up to three mismatches with the references sequences were allowed. Reads represented with less than 2% were excluded from the figure.

Overview of the read alignments for the different pre-miH12 and pre-miSNP50 reference sequences is presented in (**Figure 6**). There was a large difference in the pri-miH12 and pri-miSNP50 processing pattern originating from different scaffolds. First, miH12-101, miH12-122, miH12-155, miH12-451, miSNP50C-451, and miSNP50T-451 scaffolds were processed to yield almost exclusively the guide strand with absent or very low concentrations of the passenger strand. The ratio between the guide and passenger strand was lower for the other miH12 scaffolds making them less attractive for future studies. Second, the analysis of the sequence length and composition of the different miH12 scaffolds revealed that the guide strands from all analyzed constructs were cleaved almost precisely at the predicted Drosha sites whereas the further downstream Dicer cleavage generated variability in the sequence length (**Figure 6a**). The latter ranged from 19–31 nt depending on the scaffold. The most prevalent miH12 length was 22 nt and originated from four scaffolds miH12-1, miH12-101, miH12-122, and miH12-203. Furthermore, the length of the most abundant read from all constructs except for miH12-101, miH12-155, miH12-451, miSNP50C-451, and miSNP50T-451 corresponded to the predicted cleavage pattern obtained from miRBase release 21.

By allowing three mismatches with the reference sequence, we observed 3' end sequence modifications for all the constructs with few that reached more than 2% threshold set for our analysis. This is in accordance with previously published data on 3'end editing events in various cell lines and tissues.^31,32,33^ The miR-101 generated the least sequence heterogeneity and active passenger strand, but the most 3'end editing. The most abundant edited read accounted for 47.20% from all reads that aligned to the pre-miH12-101 reference sequence.

Notably, the read alignments of miH12 and miSNP50 expressed from the miR-451 scaffold showed different length and cleavage patterns of the most prevalent sequences. In the case of miH12-451, the most abundant read (59.98%) was 30 nt long encompassing the guide, loop and 8 nt from the passenger strand. The incorporated 21 nt miH12 sequence was detected in only 0.05% of the reads. No full-length passenger strand was detected. This cleavage pattern is typical for the non-canonical Drosha/Argonaute 2 processing mechanism. The read alignment analysis of miSNP50C-451 and miSNP50T-451 revealed a completely different picture; for both constructs the most abundant sequences were 23 nt long, representing 48.52 and 42.28% of the reads matching the reference sequences, respectively. Similarly to miH12-451, very little passenger strand was detected.

During miH12-135 processing, the guide strand sequences were generated from the 5' arm. Interestingly, embedding miSNP50C and miSNP50T sequences into the miR-135 scaffold reversed the preference for the 5' arm to serve as a guide strand and the 3' arm as a passenger strand (**Figure 6b**).

### *In vivo* silencing efficacy and pre-miH12 processing of AAV5-miH12-101, AAV5-miH12-135, and AAV5-miH12-451 vectors

**Figure 7:**
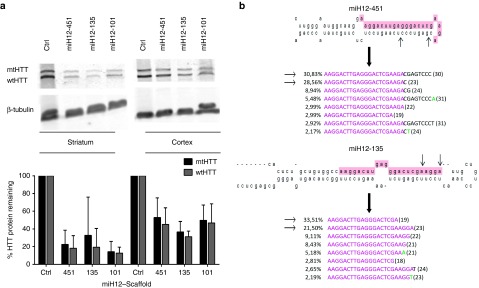
**HTT knock-down by AAV5-miH12-101, AAV5-miH12-135, AAV5-miH12-451 vectors and next-generation sequencing (NGS) analysis on miH12 processing in the humanized Hu128/21 HD mouse model**. (**a**) Top, an example western blot showing wtHTT and mtHTT knock-down by AAV5-miH12-101, AAV5-miH12-135, and AAV5-miH12-451 viruses. Bottom, quantification of HTT protein in the striata and cortices of Hu128/21 mice 2 months postinjection with AAV5-miH12-101, AAV5-miH12-135 and AAV5-miH12-451 viruses. Density of HTT bands was normalized to β-tubulin loading control and shown as a percentage of the same allele (either wtHTT or mtHTT) from Ctrl animals. (**b**) Sequence distribution (%) of reads mapping to miH12-135 and miH12-451 scaffolds. Cortices were isolated from mice 2 months postinjection of AAV5-miH12-135 and AAV5-miH12-451. RNA was isolated from tissue homogenate and small RNA NGS was performed. For each variant, the miHTT scaffold is shown on top. First two most abundant sequences are represented by the arrow. For the read alignments, up to three mismatches with the reference sequences were allowed and are shown in green. Reads represented with less than 2% were excluded from the figure.

To investigate the miH12 silencing efficacy and processing patterns *in vivo*, the humanized transgenic Hu128/21 HD mice were injected bilaterally in the striatum with 4x10^11^gc per animal of AAV5 encoding miH12-101, miH12-135, miH12-451, or Ctrl constructs. Two months postinjection, mice were sacrificed and the level of HTT silencing in the brain was evaluated by western blotting (**Figure 7a**). Similar to our *in vitro* data, miH12 induced strong more than 70% total HTT knock-down in the striatum and more than 50% HTT knock-down in the cortex. Moreover, the silencing efficiency varied between the three scaffolds with AAV5-miH12-135 showing the strongest HTT knock-down. To address the miH12 processing *in vivo*, we analyzed the prevalence and sequence composition of the guide and passenger strands by NGS. RNA was isolated from the cortices of Hu128/21 mice and small RNA NGS was performed. For each sample, we obtained between 15–30 mln small RNA reads that were subsequently adaptor-trimmed and aligned against the corresponding reference sequence. All reads shorter than 10 nt, longer than 45 nt, and represented less than 10 times were excluded from the analysis. Overview of the read alignments for pre-miH12-135 and pre-miH12-451 scaffolds is represented in (**Figure 7b**). In the case of miH12-451, the most abundant reads were 30 nt and 23 nt long, respectively. In case of miH12-135, we observed similar Dicer cleavage pattern as we observed *in vitro*. Surprisingly, the most abundant read was 19 nt long and accounted for 33.51%. We also observed similar 3'end editing events from both scaffolds compared to our *in vitro* data.

Our results demonstrate that the incorporation of identical sequences in various pri-miRNA scaffolds results in different HTT knock-down and processing of guide and passenger strands both *in vitro* and *in vivo*. As a consequence, every scaffold yielded its own “fingerprint” of mature miH12 and miSNP50 sequences. Based on the silencing efficacy and processing profile, we selected miH12-451 for further development as the artificial miRNA-based therapeutic candidate for HD.

## Discussion

Given the complexity of pathology and natural history of HD, *HTT* transcript lowering approaches come to focus since they aim to reduce the concentration of the malfunctioned protein. To date, both total and allele-specific HTT down-regulation strategies are moving forward to the clinic. siRNAs and ASOs show positive results when targeting either both alleles or only mtHTT allele *in vitro* and *in vivo.*^7,15,16,34,35^ However, the periodical readministration of these therapeutics is required which adds more risk for infections and burden on the patients. Recently, the artificial RNAi-based approach using shRNAs or miRNAs has been proposed for the sustained total and allele-selective HTT down-regulation.^10,13,18,19^ In general, shRNAs are expressed from pol III promoters and miRNAs from pol II promoters. Evidence suggests that expressing artificial shRNAs from strong pol III promoters can induce severe toxicity at high doses.^36,37,38^ In some cases, the toxicity correlates with high production of the passenger strand, independent from the target *HTT* mRNA inhibition.^39^ Importantly, switching to miRNA-based expression system can reduce the toxic effect.^36^ Thus, a promising approach for the HD therapy is considered the pol II expression of artificial miRNAs targeting *HTT* transcripts delivered with viral vectors. Nevertheless, a detailed analysis on miRNA processing patterns, such as sequence composition, guide/passenger strand ratios, and passenger strand activity is necessary to estimate the potential cellular toxicity *in vivo*.

In the current study, we have evaluated three different approaches for HTT silencing using expressed artificial miHTTs. Total HTT knock-down was induced by targeting both wtHTT and mtHTT alleles with miH1-miH21 directed towards exon 1. The feasibility of the allele-specific silencing was evaluated by targeting the CAG repeats or heterozygous SNPs rs362331 and rs362307 in the *HTT* gene. Construct optimization of the best miH12, miSNP50C, and miSNP50T candidates has been performed by introduction of single base substitutions in the guide strand sequences, adapting the expression cassette and using different cellular pri-miRNA scaffolds. The silencing efficacy, allele selectivity, analysis of miH12, miSNP50C, and miSNP50T processing patterns, and evaluation of the passenger strand activity supported the selection of the best therapeutic candidate for further development.

Several lines of investigation suggest that sufficient inhibition of mtHTT while maintaining wtHTT at adequate levels is achievable and therapeutically beneficial.^8,10,13,40^ For instance, lentiviral delivery of shRNAs targeting both HTT alleles reduced HD-like neuropathology in the HD rat model.^10^ In another study with the transgenic HD mice, more than 70% total HTT knock-down using artificial miRNAs was efficacious with no apparent toxicity 3 months postinjection.^40^ The safety of the nonallele-selective HTT approach was also evaluated in nonhuman primates where it showed tolerance to 45% HTT knock-down.^8,13^ Additionally, from the drug manufacturing perspective, single product development and drug availability for all HD-allele carriers favor nonallele-selective HD approaches forward to the clinic. Here, we identified miH12, which induced more than 70% HTT knock-down both *in vitro* and *in vivo* as the therapeutic candidate for further development. Also, we optimized the expression cassette of miH12 to make the vector suitable for clinical use.

Despite the promising results for the total HTT knock-down, the model therapy would inhibit only the mtHTT allele while preserving wtHTT at normal levels. To achieve this, we initially designed 15 miCAG constructs with either perfect or partial complementarity to the CAG repeats. It has been shown that introducing base changes at a specific position within siRNA sequences that target CAG repeats enhanced HD allele discrimination.^23^ We applied this knowledge when generating our miCAG constructs. However, none of the constructs induced good allele selectivity. Recently, ASOs and miRNAs perfectly complementary to CAG repeats with one mismatch at position 9 showed good allele selectivity.^39,41^ Differences in the mismatch position, base composition, and expression cassette could explain the contrast in allele selectivity between theirs and our CAG-targeting constructs.

To design allele-specific HD therapy by targeting the mtHTT allele-linked SNPs, ideally, HD patients would be heterozygous for a specific SNP with one isoform linked to the mtHTT. However till now, no such SNP has been discovered. Evidence shows that a combination of targetable isoforms originating from different SNPs will be required to treat the majority of HD population.^17^ Very similarly to miCAG constructs, artificial miRNAs designed to target the HD-linked isoform T of rs362307 could not discriminate well between the two heterozygous alleles. Similar results have been shown in rodent HD models using siRNAs, shRNAs and different artificial miRNAs.^17,18,19^ Moreover, the introduction of secondary mismatches did not improve the allele specificity *in vitro*. In contrast, previously it has been shown that some siRNAs targeting the isoform T of rs362307 and carrying a secondary mismatch to the wtHTT allele, showed good allele selectivity, which might be due to differences in the effective therapeutic sequence, administration, test system, and concentration.^17^

A great portion of the Caucasian HD population is heterozygous (C/T) for rs362331 in exon 50.^3^ Here, we designed 42 different artificial miSNP50 by micro-shifting the 21 nt guide strand along the HTT target sequence that contained either C or T isoform of rs362331. In contrary to previously published data with shRNAs and siRNAs, none of the constructs induced immediate good allele selectivity.^18,20^ Very likely, the length of the guide strand, sequence composition and miRNA processing contributed to the observed differences. The allele selectivity was achieved after introducing single nucleotide substitutions at specific positions within the guide strand, as previously suggested.^26^

In the canonical processing pathway, cellular miRNAs are expressed from the genome mainly by RNA pol II as long pri-miRNAs.^42^ The pri-miRNA transcripts fold into specific stem-containing RNA scaffolds which determinate their further processing by the Microprocessor complex.^43,44,45^ Further downstream, Dicer cleaves the loop-containing precursor into a mature double-stranded miRNA structure composed of the more active guide strand and its complementary passenger strand.^46^ The main concerns when using artificial miRNAs are the off-target effects and saturation of RNAi machinery caused by events linked to cellular processing of mature miRNA strands and target similarity with other genes.^38,39,47^ Here, for the first time, we evaluated the efficacy and processing of miH12, miSNP50C, and miSNP50T from several pri-miRNA scaffolds for a single HD therapeutic. We have performed detailed NGS analysis of the artificial miRNA processing and the passenger strand activity both *in vitro* and *in vivo*, which varied depending on the scaffold. Moreover, we showed that embedding miH12, miSNP50C, and miSNP50T sequences in the same miR-135 scaffold resulted in different cellular processing of guide and passenger strands. For instance, cellular processing of miH12-135 favored production of the 5'arm over the 3'arm. However, miSNP50C-135 and miSNP50T-135 processing had reversed preferences, the 3'arm served as the predominant guide strand. Variability in the miRNA scaffold, thermodynamic properties of the duplex miRNA, 3'overhangs of the both strands could result in the observed differences.

In the noncanonical pathway, the pre-miR-451 escapes Dicer cleavage and only a 5' arm strand is active on the targets.^48,49^ The mature guide strand sequence of the miR-451 is generated by cleaving the 3'arm between 10–11 nt from the stem origin which yields a 30 nt long intermediate that is subsequently trimmed by poly(a)-specific ribonuclease (PARN) into a mature ~23 nt long active molecule.^50,51,52^ Interestingly, NGS on miH12-451 processing *in vitro* showed a 30 nt long predominant read, but for miSNP50C-451 or miSNP50T-451 the read was only 23 nt long. Moreover, miH12-451 processing *in vivo* showed equal ratios of the 30 nt and 23 nt long molecules. It has been reported that PARN-associated trimming exhibit nucleotide preferences at the 3'end of AGO2-cleaved intermediates and is not required for target silencing.^52^ Construct concentration and the cellular environment could be potential reasons for the lack of a ~23 nt long active miH12 molecules *in vitro*.

Ultimately, our NGS analysis showed 3'end miRNA editing events both *in vitro* and *in vivo*. In both cases, adenosine or uracil was added to the mature guide sequences. This resulted in a formation of several mature miH12 isoforms containing the same seed region. Previous studies report that trimming and tailing influence the stability and processing of pre-miRNAs, mature miRNAs or mRNA sequences.^53,54,55,56^ However, the exact roles of miH12 mono-uridylation and mono-adenylation still needs to be determined.

Altogether, our results highlight the necessity of careful selection and characterization of the therapeutic product both *in vitro* and *in vivo* before entering the clinic. A number of parameters need to be carefully evaluated including gene silencing efficacy, miRNA processing pattern, and induction of off-target effects.

## Materials and methods

*HTT target sequences.* Homo sapiens HD mRNA, complete cds (gb|L12392.1|HUMHDA) obtained from http://blast.ncbi.nlm.nih.gov/Blast.cgi was used to identify target sequences for the artificial miHTTs. miH1-21 targeted sequences in exon 1 (pos.185–579), miCAG constructs targeted the CAG repeats (pos.367–429), miSNP50 constructs targeted SNP rs362331 (pos.7246), and miSNP67 constructs targeted SNP rs362307 (pos.9809).

*DNA constructs.* To create the miHTT vectors, miRNA sequences targeting *HTT* transcripts were embedded into the murine pre-miR-155 backbone of pcDNA6.2-GW/EmGFP-miR (Invitrogen, Carlsbad, CA) by annealing complementary oligonucleotides (Qiagen, Valencia, CA) and ligation into the linearized pcDNA6.2 plasmid. As a negative control, pcDNA6.2-GW/EmGFP-miR-neg control (Invitrogen) was used, named Ctrl. miH12 and miSNP50 sequences were subsequently incorporated in cellular pri-miRNA scaffolds of the human miR-1–2, miR-16-1, miR-26a-1, miR-101-1, miR-122, miR-135b, miR-155, miR-203a, miR-335, and miR-451a. 200 nt 5' and 3' flanking regions were included with EcoRV and BamHI restriction sites and the complete sequences were ordered from GeneArt gene synthesis (Invitrogen). In those constructs, the CMV promoter was replaced by the CMV immediate-early enhancer fused to chicken β-actin (CAG) promoter (Inovio, Plymouth Meeting, PA).

For generation of the luciferase reporters LucHTT(wt) or LucHTT(mt), HTT exon 1 containing 19 or 73 CAG repeats respectively was linked to 83 nt from HTT exon 67 containing C/T at SNP rs362307 (pos.9809) and the fragment was cloned in the 3'UTR of the RL gene of the psiCHECK-2 vector (Promega, Madison, WI). For generation of LucSNP50C and LucSNP50T constructs, 48 nt fragment from HTT exon 50 containing C/T at SNP rs362331 (pos.7246) was cloned in the 3'UTR of the RL gene of the psiCHECK-2 vector (Promega). For different LucPassenger reporters, reverse complements of the passenger strands with 5 nt added from the Dicer cleavage sites were generated by CLC Main Workbench (version 6.6.1) and ordered (Qiagen). Annealed complementary oligonucleotides were cloned in the 3'UTR of the psiCHECK-2 vector (Promega). The mfold program (http://mfold.rna.albany.edu/?q=mfold) was used to determine the secondary structure of the RNA transcripts. The mfold structures of the oligonucleotides used in this study are listed in **Supplementary Table S1**.

*Cell culture and transfections.* The human embryonic kidney (HEK)293T cells were maintained in Dulbecco's modified Eagle's medium (Invitrogen) containing 10% fetal calf serum (Greiner, Kremsmünster, Austria), 100 U/ml penicillin and 100 U/ml streptomycin (Thermo Fisher, Waltham, MA), at 37 °C and 5% CO_2_. For luciferase assays and small RNA NGS, cells were seeded in 24- or 6-well plates at a density of 1.2*10^5^ or 0.5*10^5^ cells per well, respectively, in Dulbecco's modified Eagle's medium (Invitrogen) 1 day prior transfection. Transfections were performed with Lipofectamine 2000 reagent (Invitrogen) according to the manufacturer's instructions.

*Luciferase assays.* HEK293T cells were cotransfected with miHTT expression constructs and luciferase reporters that contain both the RL gene fused to miHTT target sequences and the FL gene. Transfected cells were assayed at 48 hours post-transfection in 100 µl 1× passive lysis buffer (Promega) by gentle rocking for 15 minutes at room temperature. The cell lysates were centrifuged for 5 minutes at 4,000 rpm and 10 µl of the supernatant was used to measure FL and RL activities with the Dual-Luciferase Reporter Assay System (Promega). Relative luciferase activity was calculated as the ratio between RL and FL activities. Allele selectivity ratio of the miSNP50 and miSNP67 variants was calculated on the IC50 given as the concentration in (ng) of miSNP needed to reach the half-maximal inhibition and dividing the IC50 of the mismatch reporter against the IC50 of the match reporter.

*RNA isolation and next-generation sequencing (NGS).* HEK293T cells were transfected with 4 µg miH12 and miSNP50 expression plasmids using Lipofectamine 2000 reagent (Invitrogen) and total RNA was isolated from cells 48 hours post-transfection using Trizol (Invitrogen) according to the manufacturer's protocol. Cortices isolated from Hu128/21 mice were tissue homogenized in Trizol (Invitrogen) using gentleMACS Dissociator (Miltenyi Biotec, Bergisch Gladbach, Germany) and total RNA was isolated from Trizol according to the manufacturer's protocol (Invitrogen). Small RNA sequencing libraries for the Illumina sequencing platform were generated using high-quality total RNA as input and the NEXTflex Small RNA Sequencing kit (Bioo Scientific, Austin, TX). Briefly, the small RNA species were subjected to ligation with 3' and 5' RNA adapters, first strand reverse transcription, and polymerase chain reaction (PCR) amplification. Sample-specific barcodes were introduced in the PCR step. The PCR products were separated on TBE-PAGE electrophoresis and the expected band around 30 bp was recovered for each sample. The resulting sequencing libraries were quantified on a BioAnalyzer (Agilent, Santa Clara, CA). The libraries were multiplexed, clustered, and sequenced on an Illumina HiSeq 2000 (TruSeq v3 chemistry) with a single-read 36 cycles sequencing protocol and indexing. The sequencing run was analyzed with the Illumina CASAVA pipeline (v1.8.2), with demultiplexing based on sample-specific barcodes. The raw sequencing data produced was processed removing the sequence reads which were of too low quality (only “passing filter” reads were selected). In total, we generated between 15–35 mln reads per sample.

*NGS data analysis.* NGS small RNA raw data sets were analyzed using the CLC Genomics Workbench 6 (Qiagen). The obtained reads were adaptor-trimmed, which decreased the average read size from ~50 bp to ~25 bp. The custom adapter sequenced used for trimming all the bases extending 5' was: GTGACTGGAGTTCCTTGGCACCCGAGAATTCCA. All reads containing ambiguity N symbols, reads shorter than 10 nt, longer than 45 nt, and reads represented less than 10 times were discarded. Next, the obtained unique small RNA reads were aligned to the references sequences of the pre-miHTT constructs (**Supplementary Table S1**) with a max. of 3 nt mismatches allowed. The percentages of reads based on the total number of reads matching the reference sequence were calculated.

*AAV5 vector production.* AAV5 vectors encoding miH12 and Ctrl were produced by a baculovirus-based AAV production system. Briefly, the miRNA constructs were synthesized and cloned in an expression vector containing the CAG promotor. The CAG-miRNA cassette was obtained by digestion with restriction enzymes HindIII and PvuI and cloned in a uniQure transfer plasmid named pVD789 in order to generate an entry plasmid. The presence of the two inverted terminal repeats (ITRs) was confirmed by restriction digestion with SmaI. The ITR-CAG-miRNA cassette was inserted in a recombinant baculovirus vector (Bac.AMT5) by homologous recombination in Sf9 cells and clones were selected by plague purification. The recombinant baculovirus containing the ITR-CAG-miRNA were further amplified till P6 in Sf+ cells and screened for the best production and stability by PCR and qPCR. To generate AAV5, Sf+ cells were triple infected with three different recombinant baculoviruses expressing the ITRs-CAG-miRNA, the replicon enzyme (rep 183) and the capsid protein (cap 765). The cells were lysed 72 hours after the triple infection and the crude lysate was treated with Benzonase (50U/ml) (Merck, Darmstadt, Germany) for 1 hour at 37 °C. AAV5 was purified on an AVB Sepharose column (GE Healthcare, Little Chalfont, UK) using an AKTA purification system (GE Healthcare) and the final concentration was determined by quantitative PCR with primers amplifying a 95bp fragment from the CAG promoter region. The final product was sequence verified by NGS.

*Mice and treatments.* Humanized transgenic HD mice expressing full-length genomic human mtHTT from the YAC128 transgene and full-length genomic human wtHTT from the BACWT transgene (Hu128/21, Amber L Southwell, the manuscript in preparation) were maintained under a 12-hour light: 12-hour dark cycle in a clean facility with free access to food and water. Experiments were performed with the approval of the animal care committee at the University of British Columbia. Two-month-old animals were injected bilaterally with AAV5-miRNAs as in ref. 57. Briefly, mice were anesthetized with isoflurane, placed into a stereotaxic frame, and the scalp prepared. An incision was made along the mid-line and burr holes drilled in both hemispheres of the skull at 0.8 mm anterior and 1.8 mm lateral to Bregma. A 5 µl Hamilton syringe loaded with 3 µl of sterile saline and 2 µl of viral solution (such that the viral solution would be injected first followed by the saline) was then lowered to mid striatum at a depth of 3.5 mm and the full 5 µl was injected at a rate of 0.5 µl/minute. The needle was left in place for additional 5 minutes and withdrawn slowly.

*HTT immunoblotting.* Two months after treatment, mice were killed with an overdose of avertin followed by cervical dislocation. Brains were removed, briefly placed on ice to increase tissue rigidity, and microdissected by region. Striata and cortices were equilibrated in RNAlater solution (Ambion) overnight at 4 °C before storage at −80 °C. Allelic HTT protein was then quantified in the striatum and cortex of three animals per condition by immunoblot as in ref. 16. Briefly, tissue was lysed in single detergent lysis buffer, total protein quantified by DC assay (Bio-Rad, Hercules, CA), and 40 µg total protein resolved on 10% low-bis acrylamide gels (200:1 acrylamide:bis). Proteins were transferred to 0.45 µm nitrocellulose membranes, which were probed for HTT (MAB2166 1:2,000, Millipore, Billerica, MA) and β-tubulin loading control (1:5,000, Sigma-Aldrich, St. Louis, MO). Secondary IR dye 800CW goat anti-mouse (1:250, Rockland, Limerick, PA) antibody and the LiCor Odyssey infrared imaging system were used to visualize proteins. The intensity of each allele of HTT was normalized to β-tubulin and then to the mean value for the same allele from Ctrl injected animals on the same membrane.

**SUPPLEMENTARY MATERIAL** **Table S1.** The mfold scaffolds of pre-miRNA sequences used in the study.
