# Supplementary material for: Design, Characterization, and Lead Selection of Therapeutic miRNAs Targeting Huntingtin for Development of Gene Therapy for Huntington's Disease
Source: Mol Ther Nucleic Acids. 2016 Mar 22;5(3):e297–. doi: 10.1038/mtna.2016.7 (PMC5014463; doi:10.1038/mtna.2016.7)
Supplement: Supplementary Table S1 [file mtna20167x1.zip › Supplementary Table 1.docx]

**Supplementary Table 1**: mfold scaffolds of pre-miRNA sequences used in the study.

(**A**) miCAG variants with the inserted guide strand sequence in pink and the nucleotide substitution in purple. (**B**) miH1-miH21 variants with the inserted guide strand sequence in pink. (**C**) miSNP50C and miSNP50T variants with the inserted guide strand sequence in pink and the SNP-matching nucleotide in green. (**D**) miSNP50C and miSNP50T -18mm variants with the inserted guide strand sequence in pink, the SNP-matching nucleotide in green and the secondary mismatch to the wtHTT in dark green. (**E**) miSNP67T variants with the inserted guide strand sequence in pink and the SNP-matching nucleotide in blue. (**F**) miSNP67T-7mm variants with the inserted guide strand sequence in pink, the SNP-matching nucleotide in blue and the secondary mismatch to the wtHTT in dark green. (**G**) miH12_Scaffolds with the predicted guide strand sequence in pink and the predicted passenger strand sequence in blue (www.mirbase.org). (**E**) miSNP50_Scaffolds with the predicted guide strand sequence in pink, the predicted passenger strand sequence in blue ([www.mirbase.org](http://www.mirbase.org)), the SNP-matching nucleotide in green and the secondary mismatch to the wtHTT in dark green.
